# Application of the qSOFA score and SIRS criteria to predict 30-day mortality in patients with suspected infection in a university hospital ward in Recife, Brazil: A retrospective cohort study

**DOI:** 10.1016/j.ijregi.2025.100567

**Published:** 2025-01-14

**Authors:** Pedro Alves da Cruz Gouveia, Nayron Veloso Resende, Sara Menezes Lima Soares, Denise Maria do Nascimento Costa, Vera Magalhães da Silveira

**Affiliations:** 1 Internal Medicine Division, Hospital das Clínicas, Federal University of Pernambuco, Recife, Brazil; 2 Tropical Medicine Department, Federal University of Pernambuco, Recife, Brazil; 3 School of Medicine, Faculdade Pernambucana de Saúde, Recife, Brazil; 4 Internal Medicine Department, Federal University of Pernambuco, Recife, Brazil

**Keywords:** Sepsis, qSOFA, SIRS, Mortality

## Abstract

*Objectives:* This study aimed to assess the accuracy of quick sequential organ failure assessment (qSOFA) and systemic inflammatory response syndrome (SIRS) in predicting 30-day mortality in patients with suspected infections in a ward environment.

*Methods:* Retrospective observational cohort study with adult patients admitted to the medical ward who began their first antibiotic regimen for a presumed infection. The qSOFA and SIRS were calculated at the time antibiotics were initiated. Multivariate logistic regression and receiver operating characteristic (ROC) curves were used to assess the predictive abilities of both scores.

*Results:* Of the 244 patients, 68 (28%) died. A qSOFA ≥2 was found in 75 (31%) patients, and 233 (95%) patients met at least two SIRS criteria. Significant associations with 30-day mortality included age over 60 years, cancer, respiratory infection, and elevated qSOFA. The discrimination of 30-day mortality using the area under the ROC curve for qSOFA was 0.68 (95% confidence interval 0.60-0.77), whereas the SIRS area under the ROC curve was 0.59 (95% confidence interval 0.51-0.67), with no significant difference between the two curves (*P* = 0.056).

*Conclusions:* qSOFA and SIRS performed poorly in predicting 30-day mortality in ward patients with suspected infections, indicating a need for better prognostic tools in these settings.

## Highlights

• Few studies have evaluated the accuracy of predicting 30-day mortality of quick sequential organ failure assessment (qSOFA) and systemic inflammatory response syndrome (SIRS) in ward patients with suspected infections.
• The area under the receiver operating characteristic curve for qSOFA was 0.68 (*P* <0.001) and the area under the receiver operating characteristic curve for SIRS was 0.59 (*P* = 0.028), with no statistical difference between the two curves.
• This research found a poor performance of qSOFA and SIRS in predicting 30-day mortality in ward settings.

## Graphical abstract

**Figure:**
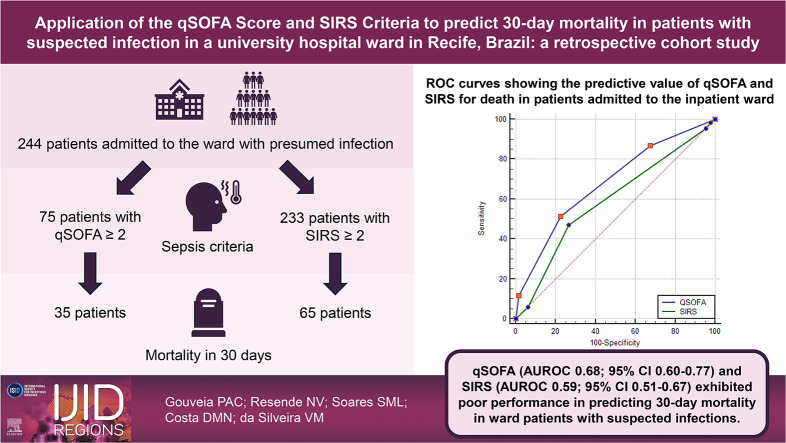

## Introduction

Early recognition and intervention are essential for the effective management of sepsis, a condition associated with high mortality rates. Health care professionals widely use the systemic inflammatory response syndrome (SIRS) criteria and the quick sequential organ failure assessment (qSOFA) score to predict prognosis and mortality in sepsis [1]. However, researchers primarily focus on patients in emergency departments (EDs) or intensive care units (ICUs) [2] while conducting limited studies on ward patients [3].

Ward patients form a distinct subgroup, often presenting with a higher prevalence of comorbidities and hospital-acquired infections than ED patients but with less severe sepsis than ICU patients [4]. Furthermore, access to ICU care is more limited in low- and middle-income countries (LMICs) than in high-income countries (HICs), making the management of sepsis in ward settings even more crucial [5]. Nevertheless, few studies evaluate the accuracy of qSOFA and SIRS in predicting mortality in ward patients, particularly, using receiver operating characteristic (ROC) curve analysis [3,[6], [7], [8], [9], [10]].

In Brazil, a middle-income country, this gap persists, with limited data on sepsis prognosis in ward settings. A prospective multicenter study in the country reported that qSOFA had low sensitivity for predicting mortality in patients in ED and wards [11]. To address this gap, the present study aimed to evaluate the prognostic accuracy of qSOFA and SIRS using ROC curve analysis in patients with suspected infection in ward settings in Brazil.

## Methods

This retrospective observational cohort study included patients aged 18 years and older who were admitted to the inpatient ward of Hospital das Clínicas da Universidade Federal de Pernambuco (HC-UFPE) between May and October 2023. HC-UFPE is a university hospital without an ED, with 12 ICU beds and 300 general beds. It admits patients from its outpatient clinic or other hospitals. We obtained patient information from medical records after the HC-UFPE Ethics Committee approved the study.

Inclusion criteria comprised inpatients starting their first antibiotic regimen for a presumed infection. We collected data when antibiotics were started to perform the qSOFA and SIRS scores. We excluded patients if antibiotics were started prophylactically or discontinued early because the health care team ruled out infection. We also excluded patients initially admitted to the ICU, pediatric, or obstetric wards. From 407 inpatient records, we excluded 163 patients, resulting in 244 patients available for analysis (Supplementary Figure S1).

We collected demographic data (age, sex, and race), comorbidities, clinical data (temperature, heart rate, respiratory rate, systolic blood pressure, and mental status), white blood cell count, and site of infection. SIRS criteria were defined by the presence of at least two of the following: temperature >38°C, heart rate >90 beats/min, respiratory rate >20 breaths/min, white blood cell count >12,000/mm³, <4000/mm³, or >10% immature neutrophils (bands). An altered qSOFA score was defined by the presence of at least two of the following: respiratory rate ≥22 breaths/min, Glasgow coma scale <15, and systolic blood pressure ≤100 mm Hg.

The primary outcome of the study was 30-day mortality. We evaluated associations between mortality and categorical variables using the Pearson chi-square test or Fisher's exact test. We applied a margin of error of 5% to all statistical tests.

We conducted a multivariate logistic regression analysis to identify factors independently associated with 30-day mortality. The variables included in the initial multivariate model were selected *a priori* based on their clinical relevance and evidence from the existing literature, among the variables with *P* <0.20 in the univariate analysis. We selected *a priori* variables for the initial multivariate model based on their clinical relevance and evidence from the existing literature, among variables with *P* <0.20 in the univariate analysis. We refined the final model using a backward elimination approach, retaining variables with a significance level of *P* <0.05. To minimize the risk of overfitting, we combined theoretical plausibility with statistical criteria in the variable selection process. The final regression model demonstrated a good fit to the data, according to the Lemeshow test (*P* = 0.96) and accurately classified death outcomes in 84% of patients.

We assessed and compared the discriminatory abilities of the qSOFA score and SIRS criteria for predicting 30-day mortality using ROC curves and the area under the ROC curve (AUROC). We calculated sensitivity, specificity, positive predictive value, and negative predictive value for qSOFA and SIRS. All analyses were performed using IBM SPSS Statistics software, version 23.

## Results

The study analyzed 244 patients, of whom 134 (55%) were women. It also identified a predominance of elderly individuals (39%), with 96 patients aged over 60 years, in agreement with Brazilian law. Patients presented a high prevalence of comorbidities (85%), with cancer affecting 77 (32%) and systemic arterial hypertension affecting 71 (29%). The most affected sites of infection included respiratory (25%) and urinary (22%) systems, and 39% of the infections were hospital-acquired. We identified a qSOFA score ≥2 in 75 (31%) patients and found that 233 (95%) met two or more SIRS criteria. Overall, 68 (28%) patients died (Supplementary Table S1).

The multivariable analysis revealed significant associations between 30-day mortality and factors such as age over 60 years, fever, cancer, respiratory infection, and altered qSOFA scores. However, the analysis did not find significant differences in the SIRS criteria in patients who died. The qSOFA score showed an adjusted odds ratio for death of 3.96 (95% confidence interval [CI] 1.73-9.07, *P* = 0.001) (Table 1).

**Table 1:** Factors associated with death within 30 days in patients admitted to the ward of a public university hospital with suspected sepsis.

	Death		Univariate		Multivariate	
	Yes N (%)	No N (%)	OR (CI 95%)	P-value	Adjusted OR (CI 95%)	P-value
Temperature						
≤38°C	21 (31.8)	88 (50.0)	1.00	0.011^a^		
>38°C	45 (68.1)	88 (50.0)	2.14 (1.19-3.92)			
Heart rate						
≤90	56 (82.3)	147 (84.4)	1.00	0.685^a^		
>90	12 (17.6)	27 (15.5)	1.17 (0.55-2.46)			
Type of infection						
Community	41 (60.3)	108 (61.4)	1.00	0.88		
Hospital	27 (39.7)	68 (38.6)	1.05 (0.59-1.85)			
Respiratory rate						
<22	25 (37.3)	117 (68.4)	1.00	<0.001^a^		
≥22	42 (62.6)	54 (31.5)	3.64 (2.02-6.57)			
Systolic blood pressure						
>100 mm Hg	23 (34.3)	79 (44.8)	1.00	0.136^a^		
≤100 mm Hg	44 (65.6)	97 (55.1)	1.56 (0.87-2.29)			
Glasgow coma scale						
15	48 (70.5)	158 (89.7)	1.00	<0.001^a^		
< 15	20 (29.4)	18 (10.2)	3.66 (1.79-7.47)			
White blood cells count						
≥4000 and ≤12,000/mm^3^	17 (26.1)	69 (39.6)	1.00	0.053^a^	1.00	0.172
<40,000 or >12,000/mm^3^	48 (73.8)	105 (60.3)	1.86 (0.98-3.49)		1.95 (0.72-5.19)	
Age group						
<60 years	27 (39.7)	121 (68.7)	1.00	<0.001^a^	1.00	0.001
≥60 years	41 (60.2)	55 (31.2)	3.34 (1.87-5.97)		3.92 (1.68-8.72)	
Cancer						
No	25 (36.7)	142 (80.6)	1.00	<0.001^a^	1.00	< 0.001
Yes	43 (63.2)	34 (19.3)	7.18 (3.87-13.34)		8.35 (3.62-21.27)	
Respiratory infection						
No	41 (65.0)	128 (75.7)	1.00	0.104^a^	1.00	0.044
Yes	22 (34.9)	41 (24.2)	1.68 (0.90-3.13)		2.46 (1.03-5.99)	
Quick sequential organ failure assessment score						
<2	33 (48.5)	136 (77.2)	1.00	< 0.001^a^	1.00	0.001
≥2	35 (51.4)	40 (22.7)	3.61 (1.99-6.52)		3.96 (1.73-9.07)	
Systemic inflammatory response syndrome score						
<2	3 (4.4)	8 (4.5)	1.00	1.000^b^		
≥2	65 (95.5)	168 (95.4)	1.03 (0.27-4.01)			

CI: confidence interval; OR: odds ratio.

a By the Pearson's chi-square test.

b By the Fisher's exact test.

Table 2 shows the performance of the SIRS criteria and qSOFA score in detecting 30-day mortality. The SIRS criteria exhibited higher sensitivity for mortality than the qSOFA score (96% vs 51%), whereas the qSOFA score had higher specificity (78% vs 4.5%). The analysis determined the qSOFA AUROC to be 0.68 (95% CI 0.60-0.77, *P* <0.001), whereas the SIRS AUROC was 0.59 (95% CI 0.51-0.67, *P* = 0.028). According to the DeLong test (*P* = 0.056), the difference between the two curves was not statistically significant.

**Table 2:** Diagnostic performances of different scores to predict 30-day mortality in patients admitted to the inpatient ward of a public university hospital with suspected sepsis.

Characteristic	Quick sequential organ failure assessment score ≥2	Systemic inflammatory response syndrome score ≥2
**Sensitivity % (95% CI)**	51.5 (36.3-66.6)	95.6 (89.4-101.8)
**Specificity % (95% CI)**	77.3 (69.4-85.2)	4.5 (0.6-08.5)
**Positive predictive value % (95% CI)**	46.7 (32.3-61.1)	27.9 (20.6-35.2)
**Negative predictive value % (95% CI)**	80.5 (72.9-88.1)	72.7 (39.2-106.3)
**Accuracy % (95% CI)**	70.1 (62.8-77.4)	29.9 (22.6-37.2)
**Area under the receiver operating characteristics curve (95% CI)**	0.68 (0.60-0.77)	0.59 (0.51-0.67)

CI: confidence interval.

## Discussion

This observational cohort study found that an altered qSOFA score was associated with a nearly four-fold increase in the risk of death in ward patients. However, despite this association, qSOFA and SIRS demonstrated poor accuracy in predicting 30-day mortality in this cohort, with an AUROC of 0.68 for qSOFA. These findings align with similar studies in LMICs, including sub-Saharan Africa, where qSOFA's accuracy reached 0.69 (AUROC) [5]. These results suggest that although qSOFA may indicate a higher mortality risk, it struggles to discriminate between survivors and non-survivors in ward settings. Another study in a ward in Brazil, focusing on patients with a high burden of HIV infection, reported AUROC values for qSOFA (0.68) and SIRS (0.69) in predicting 30-day mortality [6], a low performance with values comparable to the present study.

The differences between HICs and LMICs in health care access, infrastructure, and population profile can affect variations in the performance of mortality prediction tools. In contrast to LMICs findings, studies from HICs showed more varied results for ward patients. Studies in Italy on patients with cirrhosis [7] and Canada [8] found better qSOFA accuracy, with AUROCs of 0.78 and 0.79, respectively. Furthermore, studies conducted in Italy on geriatric patients [9], in the United States [10], and Denmark [3] reported low accuracy for qSOFA and SIRS in predicting mortality, with AUROC values very similar to those observed in this research.

Given the limited prognostic accuracy of qSOFA and SIRS, clinicians should be cautious when using these tools to predict 30-day mortality in ward patients. Although these scores may help identify high-risk patients, their poor predictive ability highlights the need for more comprehensive clinical assessments. This underscores the importance of using other tools, such as the universal vital assessment score [5], particularly, in LMICs where resources are more constrained.

This study has several limitations. First, it analyzed data from a single center in Brazil, which may limit the generalizability of the results to other settings with different health care infrastructures or patient demographics. In addition, as a retrospective study, it depended on existing medical records, which could have introduced information biases, particularly, concerning how the infection was diagnosed and the identification of the site of infection.

In conclusion, our study found that qSOFA and SIRS perform poorly in predicting 30-day mortality in ward patients starting their first antibiotic regimen for a presumed infection in Brazil. These findings align with studies from LMICs and provide valuable data to the limited literature on sepsis prognosis in ward settings. qSOFA and SIRS alone may fail to capture the full complexity of sepsis prognosis, suggesting the need for more comprehensive risk stratification tools.

## Declarations of competing interest

The authors have no competing interests to declare.

## Appendix Supplementary materials

Image, application 1

Image, application 2
