# Supplementary figures and images for: Application of the qSOFA score and SIRS criteria to predict 30-day mortality in patients with suspected infection in a university hospital ward in Recife, Brazil: A retrospective cohort study
Source: IJID Reg. 2025 Jan 14;14:100567. doi: 10.1016/j.ijregi.2025.100567 (PMC11840204; doi:10.1016/j.ijregi.2025.100567)

**Figure S1. Study Flow Chart.**


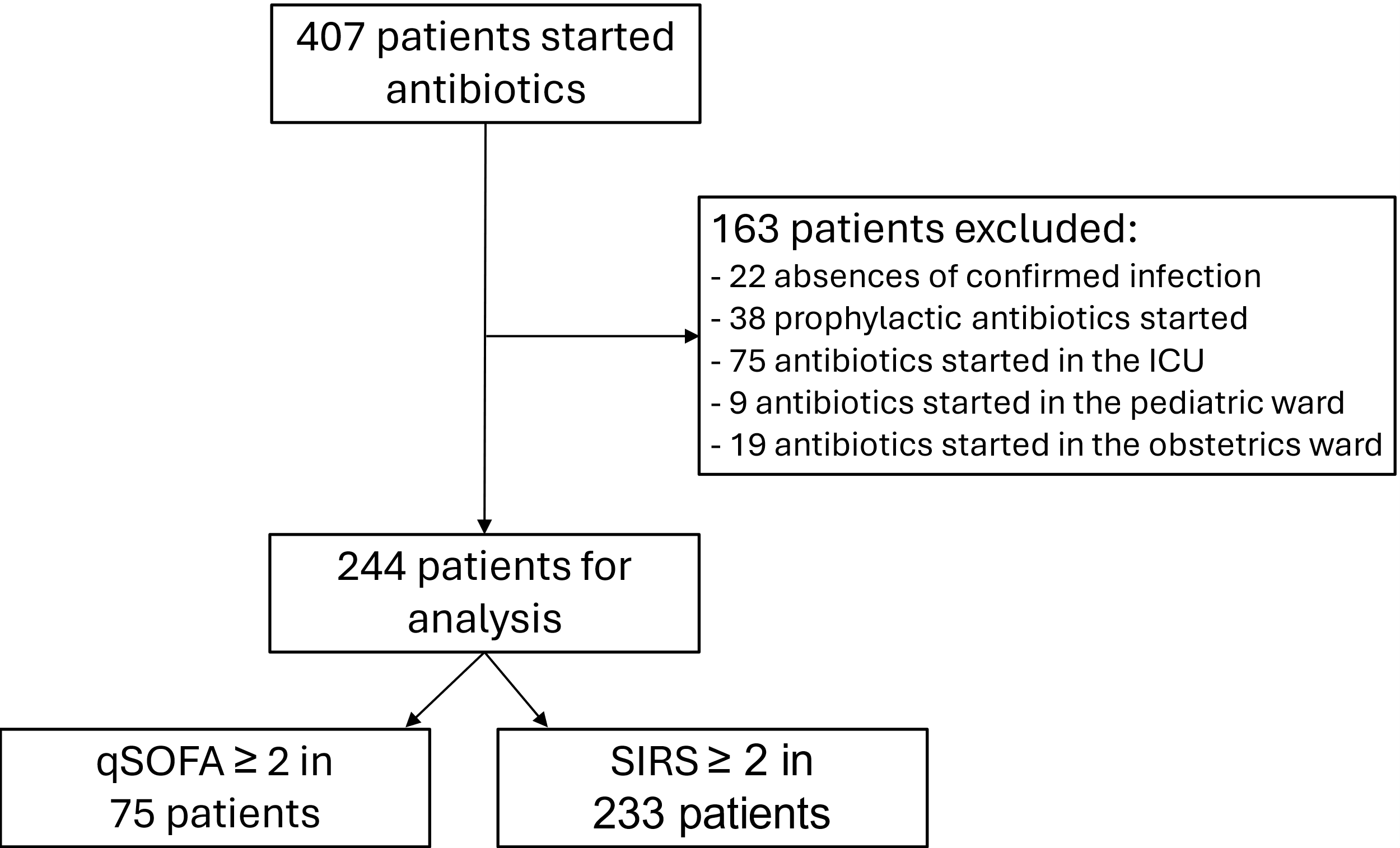

Supplement: Supplementary file 1 [file mmc1.docx]
