# Supplementary material for: Application of the qSOFA score and SIRS criteria to predict 30-day mortality in patients with suspected infection in a university hospital ward in Recife, Brazil: A retrospective cohort study
Source: IJID Reg. 2025 Jan 14;14:100567. doi: 10.1016/j.ijregi.2025.100567 (PMC11840204; doi:10.1016/j.ijregi.2025.100567)
Supplement: Supplementary file 2 [file mmc2.docx]

**Table S1. Epidemiological and clinical characteristics among patients admitted to the ward of a public university hospital with suspected sepsis.**

qSOFA: quick Sequential Organ Failure Assessment; SIRS: Systemic Inflammatory Response Syndrome

|  | Total (%)  244 (100%) | qSOFA score ≥ 2  75 (30.7%) | SIRS score ≥ 2  233 (95.5%) | p |
| --- | --- | --- | --- | --- |
| Age range  18 to 39 years  40 to 59 years  60 years or more | 76 (31.1)  72 (29.5)  96 (39.3) | 21 28.0)  19 (25.3)  35 (46.7) | 75 (32.3)  67 (28.9)  90 (38.8) | 0.483 |
| Gender  Male  Female | 110 (45.1)  134 (54.9) | 36 (48.0)  39 (52.0) | 107 (45.9)  126 (54.1) | 0.754 |
| Race  White  Multiracial  Black  Uninformed | 14 (5.7)  177 (72.5)  13 (5.3)  40 (16.4) | 4 (5.3)  55 (73.3)  2 (2.7)  14 (18.7) | 14 (6.0)  176 (75.5)  5 (2.1)  38 (16.3) | 0.953 |
| Comorbidities  Cancer  Systemic arterial hypertension  Diabetes Mellitus  Autoimmune disease  Chronic kidney disease  Others | 77 (31.6)  71 (29.1)  49 (20.1)  44 (18.0)  28 (11.5)  58 (31.4) | 40 (53.3)  19 (25.3)  12 (16.0)  14 (18.7)  2 (2.7)  59 (78.7) | 78 (33.5)  70 (30.0)  49 (21.0)  41 (17.6)  7 (3.0)  132 (56.7) | 0.016 |
| Hospital ward  Oncology  Nephrology  Internal medicine  Rheumatology  General surgery  Others  Uninformed | 43 (17.6)  38 (15.6)  32 (13.1)  23 (9.4)  20 (8.2)  85 (34.6)  2 (0.8) | 16 (21.3)  11 (14.7)  16 (21.3)  3 (4.0)  2 (2.7)  25 (33.3)  2 (2.7) | 42 (18.0)  36 (15.5)  49 (21.0)  23 (9.9)  2 (0.9)  77 (33.0)  4 (1.7) | 0.627 |
| Presumed site of infection  Respiratory  Urinary  Abdominal  Skin and soft parts  Others  Undetermined | 63 (25.3)  57 (22.9)  46 (18.5)  29 (11.6)  40 (16)  14 (5.6) | 27 (36.0)  16 (21.3)  14 (18.7)  7 (9.3)  8 (10.7)  3 (4.0) | 63 (27.0)  50 (21.5)  42 (18.0)  27 (11.6)  37 (15.9)  14 (6.0) | 0.716 |
| Death  Yes  No | 68 (27.9)  176 (72.1) | 35 (46.7)  40 (53.3) | 65 (27.9)  168 (72.1) | 0.003 |
